# Neuromorphic van der Waals crystals for substantial energy generation

**DOI:** 10.1038/s41467-020-20296-9

**Published:** 2021-01-04

**Authors:** Sungsoon Kim, Sangjin Choi, Hae Gon Lee, Dana Jin, Gwangmook Kim, Taehoon Kim, Joon Sang Lee, Wooyoung Shim

**Affiliations:** 1 Department of Materials Science and Engineering, Yonsei University, Seoul, 03722 Korea; 2 Center for Multi-Dimensional Materials, Yonsei University, Seoul, 03722 Korea; 3 Department of Mechanical Engineering, Yonsei University, Seoul, 03722 Korea

**Keywords:** Devices for energy harvesting, Two-dimensional materials, Nanofluidics

## Abstract

Controlling ion transport in nanofluidics is fundamental to water purification, bio-sensing, energy storage, energy conversion, and numerous other applications. For any of these, it is essential to design nanofluidic channels that are stable in the liquid phase and enable specific ions to pass. A human neuron is one such system, where electrical signals are transmitted by cation transport for high-speed communication related to neuromorphic computing. Here, we present a concept of neuro-inspired energy harvesting that uses confined van der Waals crystal and demonstrate a method to maximise the ion diffusion flux to generate an electromotive force. The confined nanochannel is robust in liquids as in neuron cells, enabling steady-state ion diffusion for hundred of hours and exhibiting ion selectivity of 95.8%, energy conversion efficiency of 41.4%, and power density of 5.26 W/m^2^. This fundamental understanding and rational design strategy can enable previously unrealisable applications of passive-type large-scale power generation.

## Abstract (web summary)

Controlling ion transport in nanofluidics is fundamental to numerous material applications but designing a material for ion selection is challenging. Here the authors report a confined van der Waals graphene oxide membrane as cation selective channel for energy generation inspired by neuron electromotive force.

## Introduction

The cell body of a neuron is enclosed by a plasma membrane, a bilayer of lipids that behave similar to two-dimensional fluids^1,2^. The hydrophobic interior of the lipid bilayer exhibits low permeability to ions. This enables cellular membranes to form barriers between the external environment, cytoplasm, and organelles. In cellular membranes, channels are ion-specific pores that permit specific types of ions, such as Na^+^, K^+^, and Ca^2+^, to flow across the membrane driven by concentration gradients. These ion channels produce a transient change in the electrical potential of the plasma membrane, called action potential^3^. These energy-efficient electrical signals are the fastest means of communication in the body, spreading over the plasma membrane at tens of metres per second and diffusing 10^7^ ions across the membrane per second^1,2^. The movement of ions through open channels controls the electrical potential across membranes so that it provides a basis for electrical signal transmission or high-speed communication between different parts of the membrane^1,2^.

**Fig. 1:**
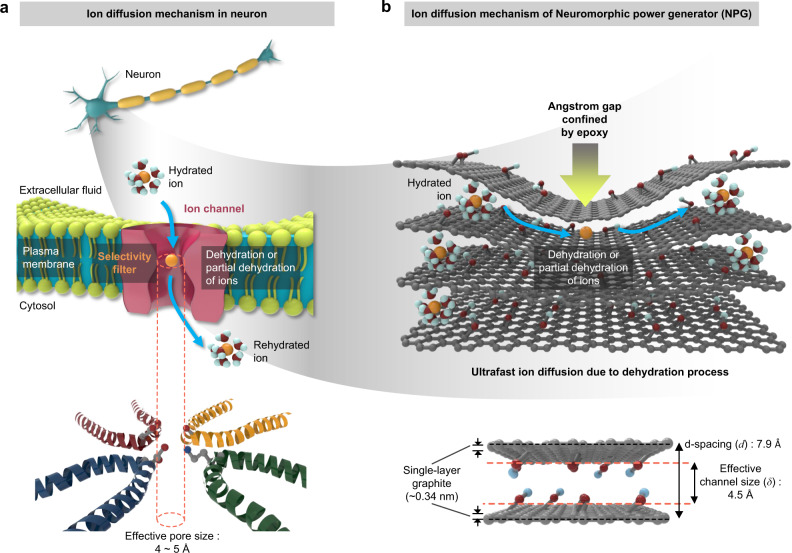
Schematics of the biological ion channel and physically confined graphene oxide. **a** Schematic of ion diffusion mechanism of biological ion channel with effective pore size. **b** Schematic of ion diffusion mechanism of physically confined graphene oxide with effective channel size.

There are several vital features critical to an ion channel for generating an electromotive force (EMF) in the neuron (Fig. 1a). First, the pore size of the channel should be similar to the size of the cation (4‒5 Å) (bottom, Fig. 1a) and smaller than that of the cation’s hydrated state^4^. This also requires the dehydration of hydrated cation in the extracellular fluid, while they pass through the channel. Second, the carboxyl group (–COOH) (negative charge) from aspartic acid and glutamic acid in the filter is required to increase the cation selectivity^4,5^ (bottom, Fig. 1a and Supplementary Fig. 1). The desired pore size with a surface charge may lower the activation energy for the dehydration of the cations. Third, the structural stability of the pore should be maintained in the liquid phase. This is achieved by the presence of an amino group (-NH_2_) from lysine, which forms the hydrogen bonding with nearby chains and prevents the pores from expanding or falling apart in the liquid phase^4,5^ (bottom, Fig. 1a and Supplementary Fig. 1). Although significant advances have been made through several selective ion-transport techniques^6–22^, including osmotic energy conversion using a two-dimensional (2D) porous structure^14,16^ or three-dimensional (3D) heterogeneous composites^12,18,19^, meeting most, if not all, of these requirements remains a challenge. In this regard, membranes with restacked two-dimensional materials, such as graphene oxide^6,8^, MXene^21^, and MoS_2_^23^, have been developed as ion-selective membranes for vertical cation flow within angstrom-sized channels. Additionally, several methods of oxidative functionalization and composite membranes of 2D material/nanofiber were developed for tuning the surface charge density, leading to an increase in ion selectivity^24,25^. Robust channels, such as a diode current-type organic membrane^12^ and a channel with organic epoxy^20^ that lasts for prolonged periods, were developed, leading to enhanced structural stability. Ideally, one would like an ion channel capable of satisfying all three of these requirements to create a technique that provides practical energy generation applications, similar to those found in a neuron channel.

Herein, inspired by the EMF generation in neuron cells by cation transfer, we demonstrate that the homogeneous confined layered channels can resemble cellular membranes in passing cations selectively. For this purpose, we prepared graphene oxide (GO) van der Waals (vdW) crystal with an effective channel height comparable to the size of cations. We simply used the epoxy to physically confine the interlayer distance of a channel for cation transfer to develop EMF (top, Fig. 1b). Similar to a neuron cell, carboxyl groups (–COOH) and hydroxyl groups (–OH) are present on the surface of GO (Supplementary Fig. 2), which can (i) lower the activation energy for the dehydration of cations and (ii) achieve cations selectivity within the Debye length through attractive interactions (bottom, Fig. 1b). However, unlike the typical layered structure, which generally expands in liquids because of the relatively weak van der Waals bonding compared to the strong hydrogen bond of an aqueous solution^26,27^ (Supplementary Fig. 3), the nanochannel size in our GO film is robust in liquids (top, Fig. 1b). This permits non-equilibrium steady-state cation diffusion for an extended period of time. With these three requirements of GO as a channel, namely, pore size, surface charge, and pore stability, we show outstanding performance metrics such as ion selectivity of 95.8%, energy conversion efficiency of 41.4%, and power density of 5.26 W/m^2^. Then, the single neuron-like EMF unit cell is integrated into cell packing for potential large-scale EMF applications.

## Results

### Ionic transport characteristics of graphene oxide confined channel

**Fig. 2:**
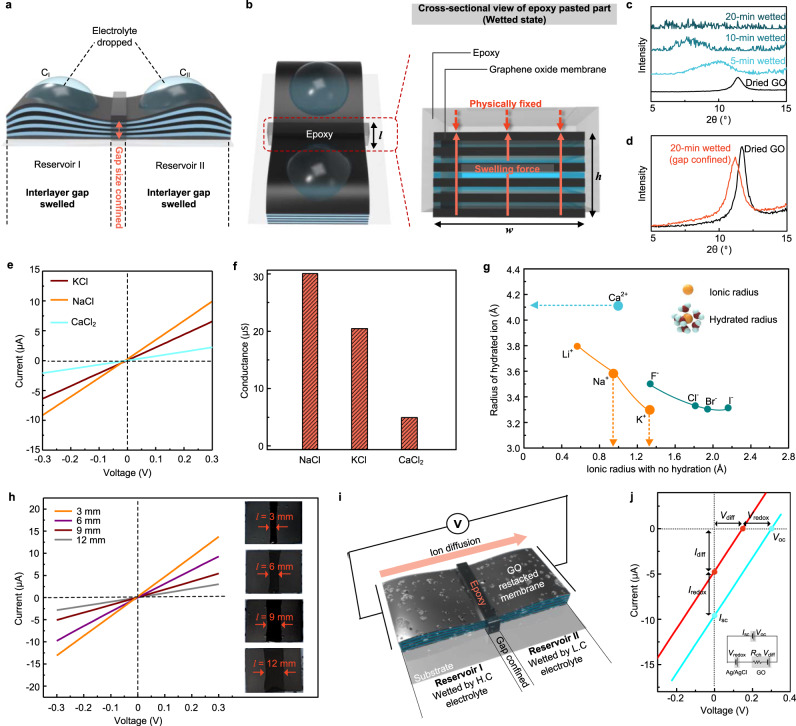
Ion transport and energy conversion. **a** Schematic of neuromorphic concentration cell. The graphene oxide membrane is divided into epoxy-pasted part (channel) and non-epoxy-pasted part (reservoir). *C*_I_ and *C*_II_ are the electrolyte concentration of reservoir I and II, respectively. **b** Schematic of physically confined channel by epoxy. *l*, *w*, and *h* are the length, width, and height of the epoxy-pasted part (channel), respectively. **c** XRD patterns when reservoir parts of GO membrane is wetted by water. **d** XRD patterns when channel part of GO membrane is wetted. **e**. *I*–*V* curve data without concentration gradient of KCl, NaCl, and CaCl_2_. The concentration of each solution is 0.1 M. **f** Ionic conductance value of GO membrane as a function of types of cation. **g** Size distribution of ions that are hydrated and not hydrated. **h** Ionic conductance of GO membrane as a function of length of epoxy-pasted part. **i** Schematic of ion diffusion direction when concentration gradient is applied. **j** *I*–*V* curve data under a concentration gradient of 10^3^ across the channel. The value by ion-selective diffusion (red line) is obtained by subtracting the redox reaction of the electrode by the unequal chloride ion concentration at the measured value (blue line). *V*_oc_, *I*_sc_*, V*_diff_, and *I*_diff_ are the open-circuit voltage, short-circuit current, diffusion voltage, and diffusion current, respectively. *V*_redox_ and *I*_redox_ are the voltage and current from redox reaction of the electrode.

To evaluate this concept, we fabricated the confined channel from the GO layer that was physically confined with epoxy. The channel connects two reservoirs (Fig. 2a). Reservoirs I and II are at two different concentrations, *C*_I_ and *C*_II_, and are connected by a cation conducting channel. When *C*_I_ > *C*_II_, the ion flows from I to II to eliminate the concentration gradient. As opposed to the expansion of the reservoirs in liquid, the cation passes selectively through the epoxy-confined channel (Fig. 2b). At this point, the channel dimensions are defined as follows, unless specified otherwise: width *w* = 1.5 cm, length *l* = 3 mm, and height *h* = 5 μm (Fig. 2b). X-ray diffraction (XRD) patterns of the two reservoirs exhibit apparent time-dependent peak variations (Fig. 2c): (i) low-angle shift because of an increase in the interlayer distance beyond 7.6 Å, (ii) broadening peaks caused by sequential increases in the interlayer distance (or separation), and (iii) no XRD peaks for reservoirs with large enough distance beyond the experimental resolution limit. The GO expands in the aqueous solution because the hydrophilic surface of GO attracts water molecules into the layers, thereby hydrating the GO layers^26^. Meanwhile, the peaks from the confined channels shift by 0.46° (Fig. 2d), corresponding to 0.3 Å. This indicates the effective channel confinement with the interlayer distance (*d*) of 7.9 Å. The free spacing (*δ*)^7^ is obtained as 4.5 Å by subtracting the GO layer thickness (3.4 Å) from *d* (Fig. 1b, Supplementary Note 1 and Supplementary Fig. 4). It is noteworthy that the free spacing of 4.5 Å in the confined channel is comparable to the ion channel size of the plasma membrane (4‒5 Å) that permits Na^+^ to pass through for communication.

To establish that only cations pass through the channel, we have characterised the ionic transport properties of the confined channel. First, we investigated the effect of the type of cation on the *I‒V* characteristics of the devices. In this case, the salt concentration in the two reservoirs is kept identical (0.1 M), and a voltage is applied across the membrane. Consequently, an ionic current through the membrane can be recorded. Figure 2e shows a representative *I‒V* response in the absence of the concentration gradient system. The Ohmic conductance *G* in the voltage increase from −0.3 V to +0.3 V for the channel of length *l *3 mm is plotted for Na, K, and Ca ion transport, where the *I‒V* is linear. *G* increased from 4.8 μS for CaCl_2_ (blue line), to 20.1 μS for KCl (red line), and to 30 μS for NaCl (yellow line), respectively (Fig. 2f). In our system, the dehydration energy barriers of divalent ions are much higher than those of monovalent ions^8,20,28^, referred to as the hydration enthalpy value of each ion (Supplementary Table. 1). For this reason, Ca^2+^ ion transport is relatively suppressed compared with the monovalent ions Na^+^ and K^+^. However, the conductance of monovalent ions reveals the size-dependent penetration of ions in the dehydrated state^29–31^. That is, smaller ions pass through the confined channel more readily under an applied electric field (Fig. 2g and Supplementary Note 2). We also investigated the effect of channel length *l* to examine the size scaling for ion transport (Fig. 2h). Channel length was controlled by using different tips when applying epoxy to the GO membrane (Supplementary Fig. 5). As anticipated, the *G* versus *l *plot for Na ion reveals that the shorter *l *exhibits the higher conductance.

Second, we produced a chemical potential gradient by setting NaCl concentrations gradients between two reservoirs, e.g., 1 M NaCl for Reservoir I and 1 mM NaCl for Reservoir II (Fig. 2i and Supplementary Fig. 6). The negatively charged channel surface of GOs and confined channel size selectively pass Na^+^, resulting in a net current flow^15,21,32^. By measuring the *I‒V* response of the confined channel in the concentration gradient system, we can measure the short-circuit current (*I*_sc_) in the absence of external bias. Meanwhile, the cell potential can be obtained from the open-circuit voltage (*V*_oc_) (blue line, Fig. 2j). The observed *I*_sc_ and *V*_oc_ originated from the (i) Na^+^ transport through the confined channel described above and (ii) thermodynamic activity difference (*a*_Cl_^−^_[I]_ and *a*_Cl_^−^_[II]_), which is related to the concentration difference between the two reservoirs (generally referred to as a concentration cell)^15,21,32^. This concentration cell can be activated using a confined channel to separate and connect two reservoirs differing in Cl^−^ concentration^15,21,33,34^. This contribution can be estimated by the Nernst equation, where standard state cell voltage is *E*° = 0 (standard Gibbs free energy *G*° = 0) and is therefore reduced to *E* = *RT*/*zF* ln(*a*_Cl_^−^_[I]_/*a*_Cl_^−^_[II]_) (Supplementary Fig. 7 and Supplementary Note 3). It corresponds to a concentration cell current *I*_redox_ ≈ 4.9 μA and a concentration cell voltage *V*_redox_ ≈ 0.16 V (Fig. 2j) with a concentration gradient of 1 M–1 mM. This concentration gradient is used unless specified otherwise. We have also explicitly modelled the effects of the Na^+^ transport and activity difference on the *I‒V* response (Supplementary Notes 3 and 4). A reasonably good agreement with the experimental results was observed. The net flow of Na^+^ is equal to the diffusion current *I*_diff_ of 4.9 μA. This Na^+^ flow produces a diffusion voltage *V*_diff_ of 0.16 V across the confined channel (red line, Fig. 2j). As shown in Supplementary Note 4, ion selectivity is calculated as the ratio of the theoretical voltage calculated by the Nernst equation to the measured voltage. It is a value for how selectively the ion diffuse, and the confined GO has excellent ion selectivity of 95.8% even at high concentration of 1 M NaCl solution. The energy conversion efficiency corresponding to the maximum power generation is calculated as shown in Supplementary Note 5. Our system shows an energy conversion efficiency value of about 41.4% together with 95.8% in ion selectivity, which is the highest reported for osmotic energy system (Supplementary Table 2). The power density (Supplementary Note 6) by the cation selective diffusion, excluding the value contributed by the redox reaction of the electrode, is calculated to be about 5.26 W/m^2^ (power density including redox reaction: 10.56 W/m^2^). We also measured the *I–V* response under concentration gradients 10^1^ and 10^2^ (Supplementary Fig. 8), exhibiting increased *I*_sc_ and *V*_oc_ with increasing concentration gradients. The cation selectivity was maintained above 90% for both the concentration gradients. The capability to convert chemical energy into electrical energy by the combination of cation transport and redox reaction, and in particular, increase in *I*_sc_ and *V*_oc_, is distinct from previous studies where ion sieving is investigated.

### Performance and stability of neuromorphic cell

**Fig. 3:**
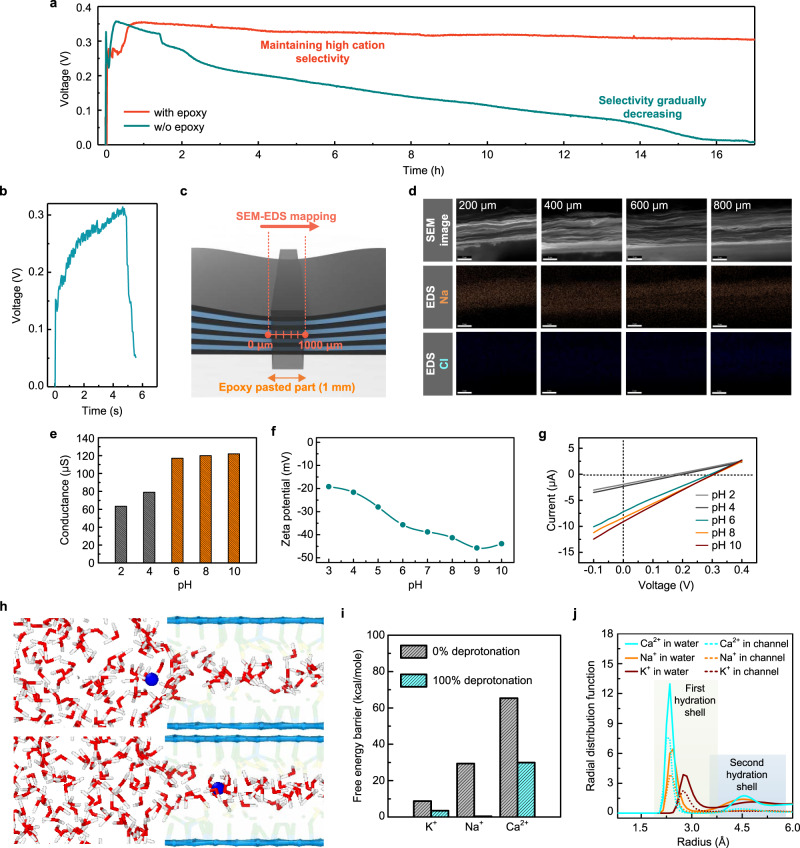
Ion mobility of sodium ions in the angstrom-scale channel. **a** Open-circuit voltage with respect to time under a concentration gradient of 10^3^. Epoxy-pasted cells maintain a more stable voltage than non-epoxy-pasted cells. **b** Open-circuit voltage data with respect to time to assess the transition from non-steady-state to steady-state diffusion. **c**. Schematic of the part on which SEM–EDS mapping analysis is applied. **d** SEM–EDS mapping results of epoxy-pasted part (1 mm). Scale bars, 2 μm. **e** Ionic conductance value of GO membrane as a function of pH at 1 M NaCl. **f** Zeta potential of GO. **g** *I–V* curve data under a concentration gradient of 10^3^ across the channel with various pH conditions. **h** The simulation showing the ion entrance into the GO channel. Carbon atoms in GO sheets are shown as blue plates, oxygen as green spheres, hydrogen in yellow, Na^+^ in blue, water oxygen in red, and water hydrogen in white. **i** Free energy barriers of ion dehydration when ions enter the GO channel. **j** Radial distribution function of oxygen atoms on water molecules around the ions in confined GO channels and bulk water.

To gain further insights into neuromorphic cell performance, we have investigated this non-equilibrium thermodynamic framework. Our neuromorphic cell can provide stable *V*_oc_ for an extended period of time and sufficient for a potential prototype EMF device. For example, Fig. 3a shows the measured voltage outputs between Reservoirs I and II bridged by the confined channel (1 mm in length) as a function of time and highlights several key points. First, the voltage output increases sharply and saturates within a few seconds at 0.32 V, which is equivalent to the sum of the voltage outputs induced by the Na^+^ transport (0.16 V) and redox potential at the Ag/AgCl electrodes from thermodynamic activity difference^15,21^ (related to concentration of Cl^−^) (0.16 V). This process can be referred to as non-steady-state diffusion. Here, the concentration (related to the voltage output) varies with time as well as location, and saturation is attained within a few seconds. Second, the voltage output of 0.32 V is likely to be maintained over a period of 18 h (the entire time of the experiment). The output voltage (open-circuit voltage) value indicates that the ion selectivity is more than 90% by the diffusion potential equation in Supplementary Note 4. This is because the size of the channel does not expand or change due to the epoxy and remains constant, and the electric double layer overlapping is maintained accordingly. We also confirmed the stability of the confined GO membrane by XRD measurements after Na^+^ passes through the channel (Supplementary Fig. 9). The XRD peak is nearly identical to that of the confined GO membrane wetted for 20 min (Fig. 2d). However, for the GO without epoxy, the output voltage value decreases over time and eventually becomes zero. We also confirmed the ion selectivity and the stability of the confined GO membrane in 100 mM/10 mM gradient conditions, which is a similar gradient to that of a biological ion channel (Supplementary Fig. 10). Thus, this stability can be considered as a result of steady-state Na^+^ diffusion inside the channel. When the cell was sealed so as not to be dry, the voltage output was maintained over 150 h. It is a long-time operation even in high concentration (1 M NaCl), which is about twice the NaCl concentration of seawater (Supplementary Fig. 11).

Further insights into the ion selectivity of the diffusion process, shown in Fig. 3a, can be obtained by examining the elemental (Na and Cl) distribution as a function of the distance *x* from the edge of Reservoir I to that of Reservoir II, after diffusion for time *t* (Fig. 3b-d). Specifically, we selected the diffusion profile at time *t* = 5 s along the distance *x* by using a scanning electron microscope (SEM) and energy-dispersive X-ray spectroscopy (EDS) (Fig. 3d). Here, it is assumed to have shifted from a non-steady-state to steady-state diffusion, based on the attainment of 0.32 V (Fig. 3b). EDS elemental mapping of Na^+^ and Cl^‒^ at the cross-sectional channel reveals that the presence of Cl^‒^ ions is not apparent, whereas that of Na^+^ is noticeable along *x* (Fig. 3d). This indicates that ion selectivity is also very high during the initial diffusion (non-steady state), and the diffusion of Na^+^ inside the channel occurs very rapidly. We used Fick’s first law to analyse the diffusion of Na^+^ into the confined channel when the concentration of Na^+^ is assumed to be held constant at the edge of Reservoir I^33^. This is a reasonable assumption because we observed that only 10^7^ out of the 10^18^ Na^+^ ions (1 M NaCl in Reservoir I considering the volume of solution) are required to flow through the channel to induce the voltage outputs of 0.16 V (see Supplementary Note 7). Thus, the ion diffusion coefficient was obtained by Fick’s first law equation (Supplementary Note 7 and Supplementary Fig. 12). The diffusion coefficient of Na ions has a value in the range of 2.56 × 10^−9^ m^2^/s–3.34 × 10^−9^ m^2^/s. It is approximately 1.75–2.3 times larger than that (1.46 × 10^−9^ m^2^/s) in the bulk state. In addition, we analysed the Na^+^ mobility in the confined channel (Supplementary Note 8 and Supplementary Fig. 13). The Na^+^ mobility was calculated as a value in the range from 8.5 × 10^−8^ m^2^/V ∙ s to 13.56 × 10^−8^ m^2^/V ∙ s. According to the Einstein relation (*μ* = *zeD*/*k*_*B*_*T*), this is consistent with the calculated diffusion coefficient value.

Figure 3e shows the conductance of the confined GO membrane at 1 M NaCl with various pH conditions. The conductance increases as pH increases, which is similar to that in the basic pH region; this indicates that the dehydration energy barrier of Na^+^ in a confined GO membrane becomes small at a high pH, and vice versa. This seems to be caused by the deprotonation degree of the carboxyl group according to the pH, which changes the surface charge density of the GO surface (Fig. 3f). Additionally, because of the pH dependence of the GO surface charges, both the open-circuit voltage and the short-circuit current increased with the pH (Fig. 3g).

To support this data, molecular dynamic (MD) simulations were performed. The simulation setup is shown in Fig. 3h, Supplementary Note 9, and Supplementary Fig. 14, where the GO channel was in contact with the reservoirs. We considered two chemical states of the GO: (i) functionalized with a pristine carboxyl group (-COOH) and (ii) deprotonated carboxyl group (-COO^−^). When the carboxyl groups are 100% deprotonated, the dehydration energy barrier decreases for K^+^, Na^+^, and Ca^2+^, but decreases the most in the case of Na^+^, and the energy barrier almost disappears (Fig. 3i). It is considered that water molecules are more easily stripped as the deprotonated carboxyl group weaken the interaction between the Na^+^ and water molecules. This simulation result is consistent with our measurements of pH-dependent conductance (Fig. 3e). It is known that certain dehydrated ions inside the confined region have higher ionic mobility and permeability than fully hydrated ions^28,30^. Therefore, if the energy barrier of dehydration can be reduced, the mobility of the dehydrated ions inside the channel can be increased. However, it is worth noting that a high number of functional groups increases the activation energy required for ion transport^30^. This could be the reason that the diffusion current value does not increase as much with increasing pH within the basic region (Fig. 3g).

Figure 3j shows the radial distribution function of the oxygen atoms in the water molecules surrounding the ions. The oxygen density indicates the amount of water surrounding the ions. Assuming that the ions in the bulk water are fully hydrated, the ions are partially dehydrated in the confined GO channel, which could be confirmed by the reduction of the oxygen atom density peaks. In the confined GO channel, the second hydration shell of Na^+^ almost disappears. This indicates that partially dehydrated Na^+^ is smaller than K^+^, which is consistent with our experimental results (Fig. 2e and f). We note that the high-diffusion kinetics of Na^+^ in the confined channel can be attributed to the effective dehydration of Na^+^ ions by the carboxyl functional group, which increases ion mobility^31,35^ (Supplementary Note 2).

### Characteristics of neuromorphic cell under various humidity conditions

**Fig. 4:**
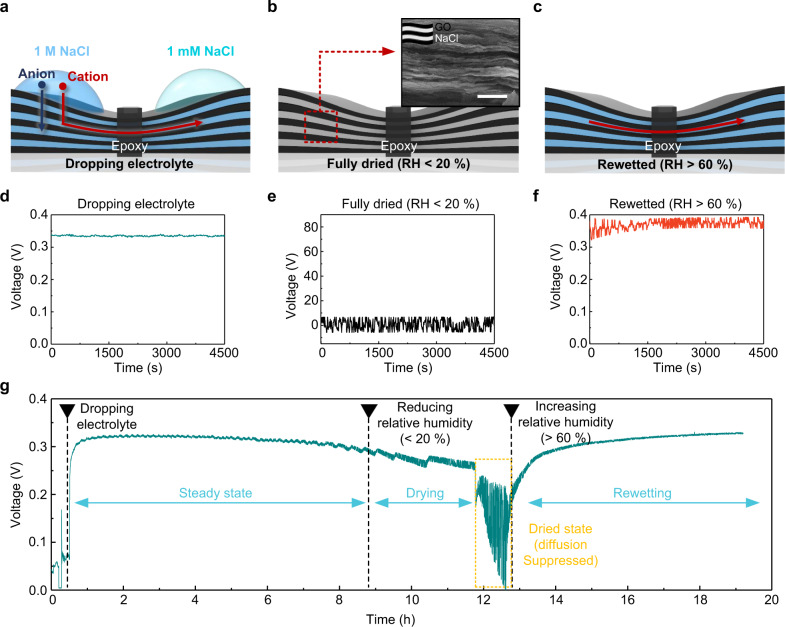
Humidiy dependence of neuromorphic cell. **a** Schematic of a cell with a drop of NaCl solution. **b** Schematic of a fully dried cell. Inset: SEM image of fully dried GO (non-epoxy-pasted part). Scale bar, 3 μm. **c** Schematic of a cell wetted again at relative humidity above 60%. Open-circuit voltage data over time when **d** NaCl solution was dropped on a cell, **e** a cell was fully dried, **f** a cell was rewetted at relative humidity above 60%. **g** Consecutive open-circuit voltage data for 20 h under varying humidity.

For real application of our neuron-like cell, e.g., portable EMF device, a cell in a dry state is desirable, i.e., the liquid should be removed entirely. Considering this, we investigated the potential applicability of our neuron-like cell to a non-solution environment, which is the dry cell. We studied the three states to achieve cell operation in ambient humidity. Humidity was controlled using a custom-designed setup^36^ (Supplementary Fig. 15). First, when a NaCl solution with a different concentration is dropped on each side of the reservoir (Fig. 4a), the water as a medium carries Na^+^ effectively through the confined channel. This yields an output voltage of 0.32 V (Fig. 4d). Second, when the water in both the reservoirs is dried completely (Fig. 4b), Na^+^ transport ceases, resulting in the suspension of output voltage (Fig. 4e). SEM–EDS mapping results (Supplementary Fig. 16) of the reservoir part in the completely dried cell indicates that the reservoir part of the GO membrane contains NaCl between the interlayers. Third, although the water appears to fill in the confined channel when the relative humidity (RH) increases to 60%, but it does not cover the outer surface of the channel (Fig. 4c) and therefore, restores the output voltage to 0.32 V (Fig. 4f). This phenomenon can be explained by heterogeneous nucleation of the water that catalyses the liquefaction in the confined channel as follows.

In general, water could condense when the supersaturation of water vapour functions as a driving force^37^. A value of *p*/*p*_sat_ less than one implies that supersaturation of water vapour does not occur, which in turn prevents condensation of water (Supplementary Note 10). However, condensation of water occurs even when the vapour is not supersaturated. This is called capillary condensation. In the GO channel, the liquid and vapour interface exhibits an inherent negative radius of curvature. This yields r* of 2.5 nm at *p*/*p*_sat_ = 0.6, and the minimum required channel height is ~3.47 nm (Supplementary Note 10). However, the critical Gibbs free energy, ΔG*, is strongly influenced by the wetting that occurs at the GO surface and catalyses the water nucleation. Given the experimental measurements of *θ* ≈ 30° (Supplementary Fig. 17 and Supplementary Note 11), which is in agreement with other studies^38,39^, *f*(*θ*) is 0.018. This yields ΔG* ≈ 10^−18^ J. This phenomenon can be explained conveniently by the Kelvin equation as well^40,41^. The Kelvin equation is expressed as

1 $${\mathrm{ln}}(p_{\mathrm{v}}/p_{{\mathrm{sat}}}) = 2V\gamma _{lv}/RT \cdot r$$

Here, *p*_*v*_ is the equilibrium vapour pressure of a negatively curved nucleus, and *p*_sat_ is the saturation vapour pressure. Capillary condensation is a special case where condensation occurs under saturation vapour pressure even among heterogeneous nucleation. This condensation is feasible without the supersaturation of vapour because the resulting liquid nucleus has a negative radius of curvature in hydrophilic nano-sized pores^40,41^. As shown in Supplementary Fig. 18, having a negative radius of curvature results in an equilibrium vapour pressure lower than the equilibrium vapour pressure when the liquid–vapour interface is flat (saturation vapour pressure). This is the reason that water condensation occurs below the saturation vapour pressure in the GO membrane^42,43^.

Figure 4g shows consecutive *V*_oc_ data for 20 h as the degree of wetting of the cell is varied: fully fluidic (Stage I), fully dried (Stage II), and partial wetted in ambient air with RH of 60% (Stage III). The voltage output level under fully fluidic condition is maintained at 0.32 V (Stage I) and decreases under drying condition at RH of less than 20% for 5 h (Stage II). It is apparent that as the humidity increases to 60% RH, non-steady-state Na^+^ diffusion occurs as the water gradually nucleates and accumulates in the confined channel and then attains the maximum output voltage in the steady-state Na^+^ diffusion process (Stage III). The water adsorption rate of the GO membrane is extremely fast, as the water uptake amount is saturated within a few minutes in the 0.6 *PP*_*0*_^−1^ environments (Supplementary Fig. 19). Because of the hydrophilic nature of the GO, the amount of water molecules absorbed inside the membrane quickly increases, and capillary condensation occurs even under the saturation vapour pressure, enabling the diffusion of Na^+^. The capability to generate EMF in ambient environment and maintain the voltage output stability for an extended period of time (particularly, for 20 h) is distinct from results of previous studies where the duration is <1 h^6,14^. It can provide flexibility for realising cellular energy generation.

### Implementation for practical applications

**Fig. 5:**
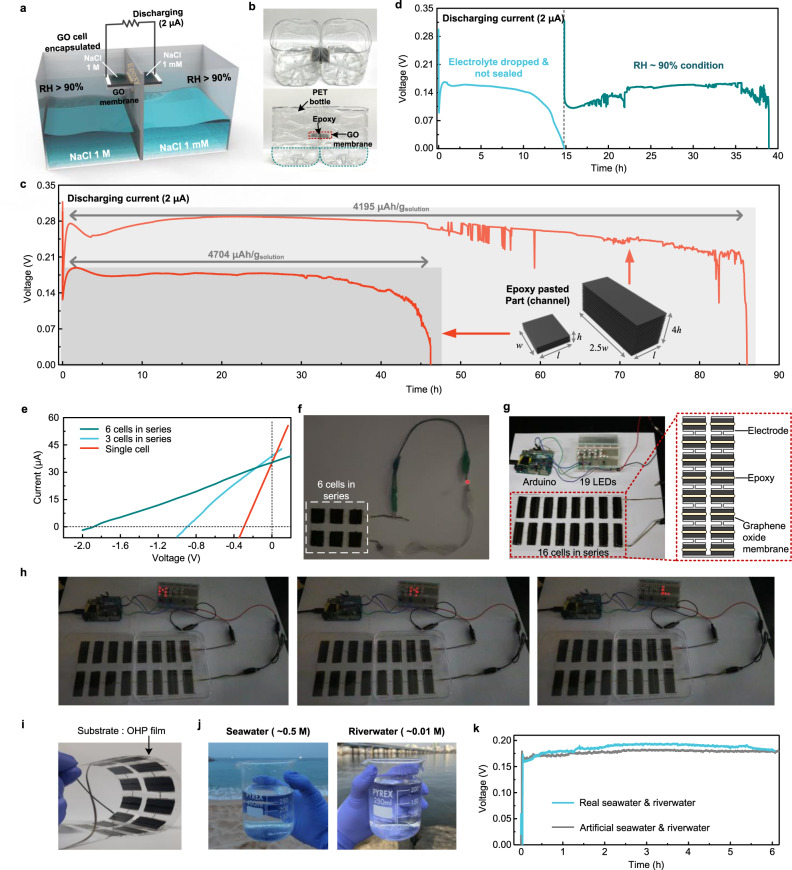
Applications of the neuromorphic cell. **a** Experimental setup for discharging test. **b** Photograph of experimental setup. **c** Discharge curve of Cell I (*w* = 1.5 cm, *l* = 3 mm, and *h* = 5 μm) and Cell II (*w* = 4 cm, *l* = 3 mm, and *h* = 20 μm) at sealed state. *l*, *w*, and *h* are the length, width, and height of the epoxy-pasted part (channel), respectively. **d** Discharge curve of a cell I (*w* = 1.5 cm, *l* = 3 mm, and *h* = 5 μm). When the cells is not sealed (RH ~50%), the NaCl solution dries, and the discharge terminates in ~15 h (blue curve). However, when the cell is sealed in that state, it can be discharged again (green curve). **e** *I*–*V* curve of series-connected cells (*w* = 1.5 cm, *l* = 3 mm, and *h* = 20 μm) under a concentration gradient of 1000. **f** Switching on a red LED using six series-connected cells. **g** Photograph of 19 red LEDs is connected to a programmed Arduino (left). Schematic of the 16 cells in series (right). **h** Switching on 19 red LEDs using 16 cells in series. **i** 16 cells that are drop-casted onto flexible plastic substrates (OHP film). **j** Real seawater and river water for application to the cell. **k** Open-circuit voltage for 6 h using concentration gradient between seawater and river water.

We have also explored the potential of the neuromorphic cell to realise practical EMF devices. As shown schematically in Fig. 5a, we considered the discharging process at a constant current in single-cells, as shown in Fig. 5b, with different channel widths and heights. At a discharge current of 2 μA, two representative single-cells exhibited a stable discharge curve and a specific capacity of >4000 μAh/g (Fig. 5c): the one with *w* = 1.5 cm, *l* = 3 mm, and *h* = 5 μm (Cell I), and the other with *w* = 4 cm, *l* = 3 mm, and *h* = 20 μm (Cell II). Note that if the output is unstable due to low ion selectivity, the discharging test cannot be performed with a constant current value. The highest energy density was obtained in cell II and was about 1180 μWh/g. In addition, the concept of the dry cell was reproduced, as described in Fig. 4g, upon discharge: fully wet (sky-blue curve) followed by dry and an RH of 90% for Cell I (green curve, Fig. 5d). At a discharge current of 2 μA, this cell exhibited 1553 μAh/g, albeit with marginal voltage output (0.16 V). The discharge voltage can be improved to >0.25 V by increasing the single-cell dimension (as in Cell II) and specific capacity (orange curve, Fig. 5c). The enhanced voltage and specific capacity originate from the increased cross-sectional area, which is equivalent to that of the cells connected in parallel.

Finally, to extend the applicability to powering devices, we evaluated the performance of the cells connected in series. For example, in series connection, *V*_oc_ was 0.89 (three-cells) and 1.9 V (six cells), a linear increase from the single-cell *V*_oc_ of 0.32 V. Meanwhile, *I*_sc_ is maintained as 33.4 μA (Fig. 5e). The six cells in series (*w* = 1.5 cm, *l* = 3 mm, and *h* = 20 μm for single-cell) delivers power to switch on an light-emitting diode ((LED)); (Fig. 5f and Supplementary Movie 1). Subsequently, we increased the cell size. *I*_sc_ increases consistently as the size of the channel increases (Supplementary Fig. 20). The 16 cells in series (*w* = 4 cm, *l* = 3 mm, and *h* = 20 μm for single-cell) was used to switch on 19 red LEDs connected in parallel and programmed by Arduino (Fig. 5g, h, and Supplementary Movie 2). The brightness of the LEDs was not significantly different from that of the case using commercial AA batteries (Supplementary Movie 3). This packing yields *V*_oc_ of 4.5 V and *I*_sc_ of 93 μA, thus providing an overall output power of 105.75 μW. The thin-film type of cell can be integrated onto flexible plastic substrates. These synchronise their motion with that of cells (Fig. 5i) and, therefore, indicate the feasibility of developing flexible power sources. Finally, we characterised the cell performance using seawater (high concentration) and river water (low concentration), which agrees well with those observed at similar NaCl concentrations (Fig. 5j and k).

## Discussion

We have developed a novel cell-like energy generation system enabling unipolar ionic transport that is challenging to obtain for an extended period of time and in certain cases have not been observed previously in the context of powering real devices. By providing the effective channel size (4–5 Å) and carboxyl functional groups similar to the biological ion channels, ion selectivity is ideally increased with concentrations similar to or higher than that of biological ion channels. Furthermore, the ion dehydration energy barrier is lowered to allow ions to pass through the channel quickly without the energy penalty. The structural stability of the biological ion channel was also implemented to maintain these characteristics for a long time. Most studies of nanofluidics to date have focused on enhancing ionic conductivity^6,13,14,19^, sensing bio-species^44–47^, and purifying water^48–51^ within a relatively short timescale of tens of seconds or a minute, our strategy of fabricating robust GO films are simply able to operate over 150 h. We have also demonstrated that the cell packing is readily applicable for increasing output power. Despite the similarities of vdW crystal with the neuron in this study, i.e., driving force, cation selectivity, structure stability, and cation dehydration (Supplementary Fig. 21), they have structural features not in common (Supplementary Table 3). Given that the diffusion mechanism depends on its structure, e.g., knock-on diffusion in the pore of neuron^52^, the structural difference (e.g., a pore for neuron and interlayer channel for vdW crystal) still need to be addressed before such strategy finds wide practical use. By providing approaches to address key issues in the context of (i) finding a correlation between cation dehydration and surface charge, (ii) modulating a channel length for lower resistance, and (iii) fabricating the channels in a parallel fashion, a few of the barriers to the transitioning of the field of nanofluidic energy conversion from being an academic area into one wherein its tenets are applied to prototyping or production capabilities, can be lowered.

## Methods

### Preparation of graphene oxide membrane

The GO films were prepared by dropping^22^ a highly concentrated GO solution (5 g/L, Graphene Supermarket) on the substrate (slide glass and OHP film). The dropping was repeated until the height of the membrane became 5 and 20 μm for each cell. The GO film was dried at 25 °C for 24 h.

### Preparation of a unit cell

Ag/AgCl ink electrode (#011464, ALS Co., Ltd) was pasted to both ends of the prepared GO membrane. The Ag/AgCl ink electrodes were dried at 25 °C for 24 h. Commercial epoxy (S-208, DEVCON) was pasted to the centre of the GO membrane. The epoxy was also dried at 25 °C for 24 h. NaCl electrolyte was dropped on the part of the GO membrane that did not have epoxy pasted on it.

### Preparation of cells connected in series

Each unit cell was connected using an ink electrode (Ag/AgCl ink paste, #011464, ALS Co., Ltd) and arranged in series. In series connection, opposite electrodes can be arranged side-by-side, as shown in the Fig. 5i. After all the cells were connected and arranged, they were activated by dropping the NaCl electrolyte.

### Preparation of real seawater and river water

Real seawater and river water were collected in Sokcho and Seoul, Korea, respectively. The concentrations of the seawater and river water were approximately 0.5 M and 0.01 M, respectively. The collected seawater and river water was cleaned by vacuum filtration to remove impurities and sand.

### Electrochemical measurements

Discharging measurement was conducted using a WBCS3000 Battery Cycler System. A cell was packed in a PET bottle with a plastic wrap to prevent evaporation of the NaCl solution. We used a digital multimeter (Agilent 34401 A) and Picoammeter (Keithley 6487) to perform the electrical measurements. All the measurements were performed in ambient temperature (25 °C).

### Characterisation

SEM and EDS were mapped using a JEOL Ltd. JSM-7001F scanning electron microscope. XPS measurements, XRD and fourier transform infrared spectroscopy were performed using a Thermo Scientific K-alpha spectrometer with Al Kα (1486.6 eV) radiation as the X-ray source, Rigaku Ultima IV X-ray diffractometer with Cu-Kα (*λ* = 1.5418 Å) radiation (40 kV, 150 mA), and Vertex 70 (Bruker), respectively.

### Water adsorption test of GO

The relative pressure was controlled in a custom-designed setup (Supplementary Fig. 15). A microelectronic balance with an accuracy of 0.0001 g was used to measure the sample weight. The GO films were pre-heated for 10 min at 50 °C and stored in a glove box before the measurements. To measure the adsorption isotherm, all samples were kept in the chamber for at least 40 min. The sample weight was constantly inspected until no more weight change was noticed to ensure that the sample reach the adsorption equilibrium. The water adsorption rate was measured by recording the weight change of samples at 25 °C and moderate humidity of *PP*_*0*_^−1^ = 0.6 for 40 min.

## Supplementary information

Supplementary Information

Description of Additional Supplementary Files

Supplementary Movie 1

Supplementary Movie 2

Supplementary Movie 3

## Data Availability

The data that support the findings of this study are available from the corresponding author upon request.
