# Supplementary material for: Neuromorphic van der Waals crystals for substantial energy generation
Source: Nat Commun. 2021 Jan 4;12:47. doi: 10.1038/s41467-020-20296-9 (PMC7782783; doi:10.1038/s41467-020-20296-9)
Supplement: Supplementary file 1 — Supplementary Information [file 41467_2020_20296_MOESM1_ESM.pdf]

Supplementary Information

Neuromorphic van der Waals crystals

for substantial energy generation

Sungsoon Kim[1,2], Sangjin Choi[1,2], Hae Gon Lee[3], Dana Jin[1,2], Gwangmook Kim[1,2], Taehoon Kim[1,2], Joon Sang Lee[3] and Wooyoung Shim[1,2]*

[1]Department of Materials Science and Engineering, Yonsei University, Seoul 120-749, Korea.

[2]Center for Multi-Dimensional Materials, Yonsei University, Seoul 03722, Korea.

[3]Department of Mechanical Engineering, Yonsei University, Seoul 03722, Korea.

*To whom correspondence should be addressed: wshim@yonsei.ac.kr

This PDF file includes:

Supplementary Notes 1–11

Supplementary Figures 1–21

Supplementary Tables 1–3

Supplementary Note 1 | Effective channel height and single channel determination

The stacked graphene oxide (GO) sheets in the layered structure are separated by an interlayer distance (d) of ~7.9 Å. The theoretical thickness (a) of the graphene monolayer is ~0.34 nm[1], and the free space for ion transport (effective channel height, δ) is estimated as δ = (d - a) $\approx$ 4.5 Å. As shown in Supplementary Figure 4, we assumed that the single GO sheet has width and length equal to the average lateral size (t $\approx$ 2.75 µm) of the GO sheets. The cross-sectional area of the single channel[2] can be estimated as $t \times \delta$. The resulting number of channels is ~3.4×10^7 across the membrane with a total area of 4.26×10^{-8} m^2. When the ion transport in the horizontal direction occurs in a uniformly stacked GO membrane, the shortest path is assumed to be the length of the channel (l).

Supplementary Note 2 | Ion mobility

Ion mobility[3] is affected by the viscosity of the solution and the ion size. It can be expressed as

$$\mu = \frac{ze}{6\pi\eta a} \tag{1}$$

where z is the valence of the ion, e is the elementary charge, η is the viscosity of the solution, and a is the ion size, respectively. It may be anticipated that the conductivity of solution will decrease with an increasing ion size. a is the hydrodynamic radius of the ion, i.e., its effective radius in the solution considering all the H_2O molecules it carries in its hydration sphere. Thus, when the ion is dehydrated, the ion mobility can be increased.

Supplementary Note 3 | Redox potential of electrodes (V_{redox})

Suppose a cell is constructed with two identical electrodes, e.g., Ag/AgCl. Furthermore, suppose that the Ag/AgCl electrodes are exposed to different chloride ion concentrations in the electrolyte. This can be accomplished by using a charge selective membrane to separate the two sections of the electrolyte with different chloride ion concentrations.

The concentration of chloride ions is Cl^- [I] on Side I of the cell. The reaction that occurs can be expressed as

$$\mathrm{Ag}_{(s)} + \mathrm{Cl}^-_{(aq)}[\,\mathrm{I}\,] \rightarrow \mathrm{AgCl}_{(s)} + e^- \tag{2}$$

On the other side, where the chloride ion concentration is Cl^- [II], the reaction is

$$\mathrm{AgCl}_{(s)} + e^- \rightarrow \mathrm{Ag}_{(s)} + \mathrm{Cl}^-_{(aq)}[\,\mathrm{II}\,] \tag{3}$$

The overall reaction is

$$\mathrm{Cl}^-_{(aq)}[\,\mathrm{I}\,] \rightarrow \mathrm{Cl}^-_{(aq)}[\,\mathrm{II}\,] \tag{4}$$

Because the standard state for the reaction is identical for both the sides,

$$\Delta G^0 = 0; \quad E^0 = 0 \tag{5}$$

Thus, the Nernst equation is reduced to

$$V_{\text{redox}} = \frac{RT}{zF} \ln \frac{a_{\text{Cl}^-_{(aq)}[\text{I}]}}{a_{\text{Cl}^-_{(aq)}[\text{II}]}} \tag{6}$$

The reaction illustrated above originates from the unequal chloride concentrations at the two Ag/AgCl electrodes. Therefore, the V_{diff} value by selective ion diffusion is obtained by subtracting the electrode reactions from the measured open circuit voltage. It is apparent that the voltage value according to the concentration calculated by the derived Nernst equation agrees well with the experimental value shown in Supplementary Figure 7.

Supplementary Note 4 | Diffusion potential by the ion selective channel (V_{diff})

As the negatively charged GO membrane is cation-selective, it can transport cations (Na^+) preferentially from the high concentration side to the low concentration side, generating the diffusion potential (V_{diff}). V_{diff} originates from the ion selectivity of the GO membrane, which can result in differences in the diffusive fluxes of anions and cations. V_{diff} can be expressed as

$$V_{\text{diff}} = (t_+ - t_-) \frac{RT}{F} \ln \left[\frac{a_{\text{high}}}{a_{\text{low}}}\right] \tag{7}$$

where t_+ and t_- are the transference numbers for cation and anion, respectively. R, T, and F are the universal gas constant, absolute temperature, and Faraday constant, respectively. a_{high} and a_{low} are the activities of NaCl in the high concentration and low concentration sides, respectively. In the above equation, (t_+ - t_-) is called ion selectivity. The charge selectivity of a GO membrane is very high (close to 0.95).

Supplementary Note 5 | Energy conversion efficiency

The membrane ion selectivity α can be calculated as,

$$\alpha = (t_+ - t_-) \times 100\% = \frac{V_{\text{meas}}}{V_{\text{theo}}} \times 100\% \tag{8}$$

where V_{meas} is the measured diffusion potential, and V_{theo} is the theoretical diffusion potential assuming a 100% charge selectivity, calculated from the Nernst equation. The energy conversion efficiency is calculated as,

$$\eta = \frac{(t_+ - t_-)^2}{2} \times 100\% \tag{9}$$

The energy conversion efficiency for the GO membrane was calculated as 41.4 %.

Supplementary Note 6 | Power density

Based on the I_{diff} and V_{diff} obtained from the measurement, the maximum output power density can be calculated. The maximum power density is obtained at half the

maximum output voltage (V_{diff}). Therefore, the maximum power density can be expressed as,

$$P_{\text{max}} = \frac{1}{4}\frac{V_{\text{diff}}^2}{R_{\text{ch}}} \quad (10)$$

Supplementary Note 7 | Diffusion coefficient (Fick's first law)

Suppose that there are cation-selective channels in the middle when ion diffusion occurs owing to the concentration gradient. Because the rate of diffusion of cations is higher than that of anions, excess cations are present in the lower concentrations, and excess anions are present in the higher concentrations. In this case, because negative charges and positive charges are separated based on the central channel, the resulting electric field strength can be calculated as follows:

$$\vec{\mathbf{E}} = \frac{xq}{4\pi\varepsilon\varepsilon_o d^2} \quad (11)$$

where x, q, ε, ε_o, and d are the number of ions, elementary charge, relative permittivity of water, vacuum permittivity, and channel length, respectively. When the cation-selective channel is ideal, the electric field strength should be as follows to form a voltage of 0.16 V at a 1000-fold concentration gradient:

$$\vec{\mathbf{E}} = \frac{V}{d} = 53\ \text{V/m} \quad (12)$$

That is, 2.61×10^7 ions must diffuse to develop 0.16 V. This is significantly less than the number of sodium ions present in 10^{-5} L of 1 M NaCl (6.02×10^{18} sodium ions).

Using the diffusion current data (Supplementary Fig. 12), the diffusion coefficient of sodium ions in an angstrom-scale channel can be calculated by Fick's first law in the steady-state region where the concentration profile does not change with time. Fick's first law equation can be expressed as

$$D = \frac{J \times L}{\Delta C} \quad (13)$$

where D, J, L, and ΔC are the diffusion coefficient, ion flux, length of the channel, and concentration, respectively. The ion flux, J, can be calculated by (($I_{\text{diff}} \times 6.02 \times 10^{18}$) / A). Here, A is the cross-sectional surface area of the channel. The diffusion current was measured by the I–V curve under a concentration gradient of 10^3 by repeating on 5 different sets of GO membranes (Supplementary Figure 12). The diffusion current value ranges from 3.5 to 4.5 μA at pH 7. As a result, the diffusion coefficient of Na ions has a value in the range of 2.56×10^{-9} m^2/s–3.34×10^{-9} m^2/s.

Supplementary Note 8 | Ionic conductance measurement and calculation for ion mobility.

The ionic conductance of the membrane is deduced from the slope of the I–V curves. The I–V data was obtained by measuring the current while varying the voltage from

−0.3 V to 0.3 V and dropping solutions of identical concentrations on both sides of the membrane. The conductance measurement data (Supplementary Figure 13b, red curve) with various concentrations reveal the surface charge-governed transport behavior. The data points at low concentrations are significantly higher than that on the blue dotted line (Supplementary Figure 13b, blue dotted line). It is apparent that the blue dotted line and the red curve converge at high concentrations. This implies that the increase in conductance owing to the increase in concentration is more dominant than that owing to the surface charge. At this point, we can calculate the ratio of the mobility of the cations to that of anions using the Hendernson equation and derive the sum of the mobilities of cations and anions from the conductance value at a high concentration (1 M NaCl). Note that the surface charge contribution could be neglected in a high concentration. The Henderson equation[4] is expressed as

$$\frac{\mu^+}{\mu^-} = -\frac{z_+}{z_-}\frac{\ln(\Delta) - z_- F E_m/RT}{\ln(\Delta) + z_+ F E_m/RT} \tag{14}$$

where μ^+/μ^-, z_+, and z_- are the mobility ratio of cation to anion, and the valences of the cations and anions, respectively. F is the Faraday constant, R is the universal gas constant, T is the absolute temperature, Δ is the concentration gradient, and E_m is the diffusion potential (V_{diff}). We determined μ^+/μ^- using the Henderson equation to be 19.31. At a high concentration, the measured value of conductance converges with the conductance of the channel without a surface charge. The conductance can be described as

$$G = F(\mu^+ + \mu^-)c\frac{wh}{l} \tag{15}$$

where c, w, h, and l are the concentration of the NaCl solution, and the width, height, and length of the single channel, respectively. From the two equations, the ion mobility of Na^+ and Cl^- could be obtained.

Supplementary Note 9 | Molecular dynamics simulation

To obtain the atomic-level insight of ions intercalation into the GO channels, molecular dynamics (MD) simulations were conducted. The ion permeation through the GO was simulated in the system shown in Supplementary Fig. 14a. Period boundary conditions were used on all simulation dimensions. The GO membrane was placed in the middle of two water chambers. The GO membrane contained four GO sheets of size 62 × 34 Å^2. The left-hand chamber was filled with saline water, whereas the right-hand chamber was pure water. The saline water was diluted by the 1.0 M of NaCl, KCl, and CaCl2 solutions. These solution concentrations in both chambers reflected the measured experimental values. To prevent free migration of the solution across the periodic boundaries, the graphene walls were placed on the right side of the right chamber and left side of the left chamber. Additionally, there was an sufficient empty vacuum space on each side of the chamber, to avoid the interaction between the reflecting periodic chambers along the x-axis boundary. The GO was functionalized with hydroxyl (–OH), carbonyl (C=O), and carboxyl (–COOH) functional groups

randomly distributed on both sides of the sheet (Supplementary Fig. 14b). The oxidization concentrations of the GO sheet, defined as the number of oxygen atoms divided by the carbon atoms on the graphene sheet, were assigned to 30 % based on experimentally crystallographic data. Furthermore, the ratio of each functional group (hydroxyl: carbonyl: carboxyl) was set as 2.52: 2.67: 1. To consider the effect of deprotonating the carboxyl (–COO), two chemical states of the GO were considered: functionalized with a pristine carboxylic group (–COOH) and a deprotonated carboxyl group (–COO).

All simulations were carried out using the Large-scale Atomic/Molecular Massively Parallel Simulator (LAMMPS) package[5]. The reactive force field (ReaxFF) was adopted to describe the potential energy of the GO membrane[6]. This force field has been known to provide accurate modeling of hydrocarbons nanostructures, taking into account possible bond formation and dissociation of different bond orders and charge polarization within the molecules. The saline water and GO interactions were calculated using the Lennard–Jones 6–12 potential[7]. The Lorentz−Berthelot mixing rule for the L–J interactions between different particles was employed[8]. The van der Waals interactions were truncated at 1.0 nm, and the long-range electrostatic interactions are computed by utilizing the particle-particle particle-mesh algorithm (PPPM)[9]. The temperature was controlled using the Nose–Hoover thermostat at 300K employing damping constants of 10 fs in the NVT ensemble. The simulation time step was of 1 fs, which was adequate to investigate the phenomena under the imposed conditions. The extended simple point charge (SPC/E) water model was employed. To reduce high-frequency vibrations of the hydrogen bonding, the SHAKE algorithm was applied to the bonds and angle of the water molecules. Using the conjugate gradient (CG) algorithm, the system energy was first minimized. After the systems approached the equilibrium state, a production run was performed for 1 ns to collect data for analysis. The post-processing was carried out using the Open Visualization Tool (OVITO)[10].

Free energy barriers

To obtain the energy barriers for ion permeation into the GO channel, we calculated the potential mean force (PMF) based on steered molecular dynamics (SMD). PMF is defined by

$$PMF(\mathrm{x}) = \int_{x_0}^{x} \langle F(x') \rangle dx' \tag{16}$$

where *F(x)* is the force acting on the ions at location x and x_0 is the reference position. To calculate the PMF of an ion, a harmonic bias potential is attached to a single dissolved ion, with equilibrium position at a point along the x axis. The harmonic potential is described as $U = k(x - x_0)^2/2$, where k is the spring constant of 100 kcal/mol·Å^2, and x_0 is the equilibrium position. The end of the spring moves with a velocity of 0.0001 Å/fs for reversible pulling. Therefore, the ion is dragged by this virtual spring, collecting resistance information that the ions experience during entrance into the GO channel. These PMF profiles have shown to emerge near the entrance of the membrane, because this insertion is energetically unfavorable for ions.

The author defined the amount of sudden increase in the PMF profile as a free energy barrier, which is the minimum energy required for the entrance of ions into the GO channel.

Radial distribution function

The radiation distribution function (RDF), denoted as *g(r)* in the equation, defines the probability distribution of finding a particle at a distance r from another tagged particle. This measurement is widely used to characterize the packing structure and includes information on the correlation between the long-distance particles.

$$n(r') = 4\pi\rho \int_0^{r'} g(r) r^2 dr \quad (17)$$

where *g(r)* is the RDF, and ρ is the number density. The coordination number indicates the number of molecules found in the range of each coordination sphere.

Supplementary Note 10 | Capillary condensation

Capillary condensation[11] is the phenomenon of condensation of materials in the vapor phase to the liquid phase, in porous media. The largest singularity of capillary condensation is that condensation in the vapor phase occurs below the saturation vapor pressure. The variation in Gibbs free energy when condensation occurs is expressed as,

$$\Delta G_r = 4\pi r^2 \gamma_{lv} - \frac{4\pi r^3}{3}\frac{1}{V} RT \ln\left(\frac{p}{p_{\mathrm{sat}}}\right) \quad (18)$$

where r, γ_{lv}, V, p, and p_{sat} are the radius of the nuclei, water–vapor interface energy, molar volume, vapor pressure, and saturation vapor pressure, respectively. A viable nucleus is one with a radius larger than or equal to the critical radius r^* ($= -2\gamma_{lv} / \Delta G_v$). Here, ΔG_v is the Gibbs free energy variation per unit volume and is equal to

$$\Delta G_v = -\frac{4\pi r^3}{3}\frac{1}{V} RT \ln\left(\frac{p}{p_{\mathrm{sat}}}\right) \quad (19)$$

In general, no condensation occurs when p is lower than p_{sat}. However, in hydrophilic microporous media, condensation can occur as the radius of curvature of the liquid phase becomes negative. The pore size for capillary condensation can be calculated using the critical radius derived from the above equation. The pore size for capillary condensation is expressed as

$$d = r^* \cos\theta = -\frac{2\gamma_{lv}}{\Delta G_v}\cos\theta = -\frac{2\gamma_{lv}}{\frac{1}{V}RT\ln\left(\frac{p}{p_{\mathrm{sat}}}\right)}\cos\theta \quad (20)$$

where r^* and θ are the critical radius of curvature of the liquid phase and contact angle, respectively. The critical Gibbs free energy can be expressed as

$$\Delta G^* = \frac{16}{3}\frac{\pi\gamma_{lv}}{\Delta G_v^2} f(\theta) \quad (21)$$

Here, $f(\theta)$ is related to the contact angle, which is equals to $[(2 + \cos\theta)(1 - \cos\theta)^2 / 4]$.

Supplementary Note 11 | Hydroxyl functional group ratio

The X-ray photoelectron spectroscopy (XPS) and contact angle measurements were used to determine the proportion of the hydroxyl functional group (Supplementary Figure 17). The O/C ratio and C 1*s* peak value of the XPS measurement data can be obtained through the following relationship:

$$P_{\mathrm{GO}} \cong 2P_{\mathrm{epoxide}} + P_{\mathrm{hydroxyl}} + P_{\mathrm{carbonyl}} + P_{\mathrm{carboxyl}} \quad (22)$$

$$P_{\mathrm{oxygen}} \cong P_{\mathrm{epoxide}} + P_{\mathrm{hydroxyl}} + P_{\mathrm{carbonyl}} + P_{\mathrm{carboxyl}} \quad (23)$$

where P_{GO}, P_{oxygen}, P_{epoxide}, P_{hydroxyl}, P_{carbonyl}, and P_{carboxyl} are the ratio of the sum of the areas of the C–O and C=O peaks to the total C 1*s* peak obtained from the XPS data, O/C ratio, fraction of epoxide, and the hydroxyl, carbonyl and carboxyl groups relative to the total number of carbon atoms in GO, respectively. The P_{GO}, P_{carbonyl} and $\mathrm{P}_{\mathrm{carboxyl}}$ values can be obtained from the C 1*s* data, yielding simultaneous equations consisting of P_{epoxide} and P_{hydroxyl}. The P_{hydroxyl} value obtained from the simultaneous equation corresponds to 17%. This agrees well with the ratio of hydroxyl groups determined by the contact angle between GO and water[12].

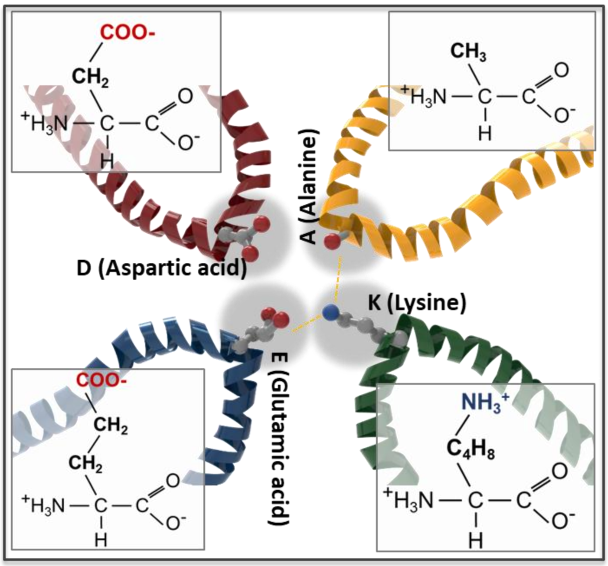

Supplementary Figure 1 | Structure of selectivity filter part of biological sodium channel. There are four amino acids in the selectivity filter. These affect the selectivity. In particular, lysine maintains the pore's structural stability by forming hydrogen bonds with its neighbours.

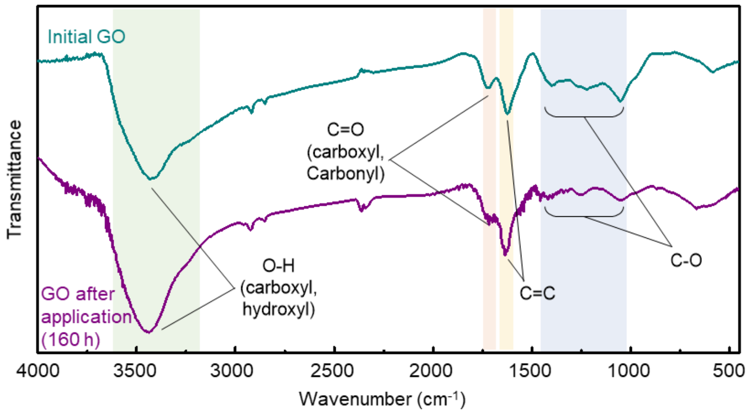

Supplementary Figure 2 | FT-IR spectra of GO membrane. The green and purple curves represent the FT-IR spectra of the GO membrane before and after application, respectively.

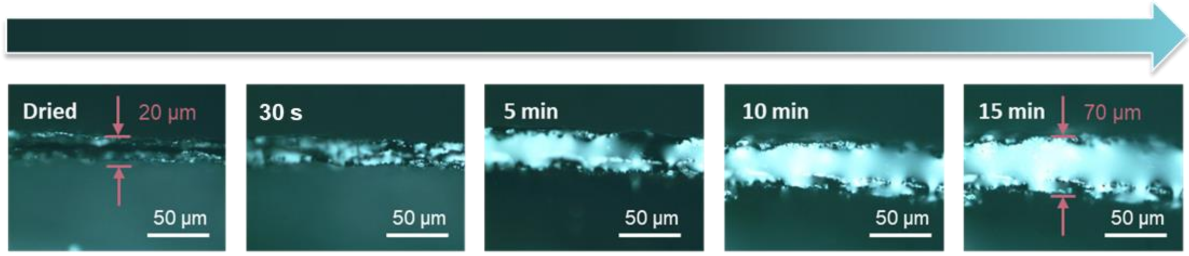

Supplementary Figure 3 | Optical microscopy image of GO membrane. GO membrane, which is in contact with water, expands over time. After 15 min, it is apparent that the thickness increased to approximately three times that of the initial state.

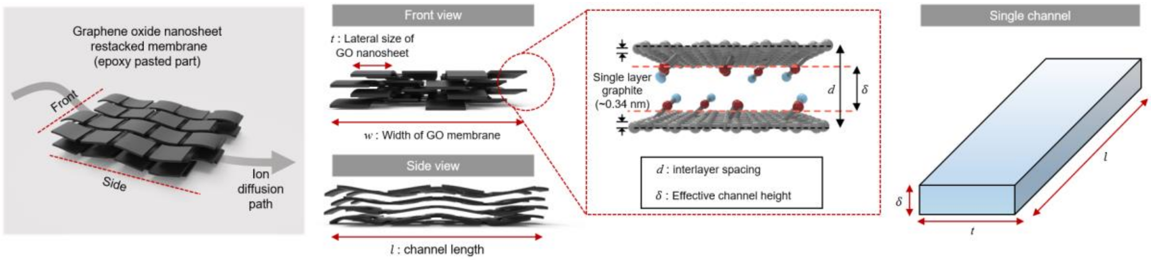

Supplementary Figure 4 | Dimensions of a confined channel (epoxy-pasted part) across the GO membrane.

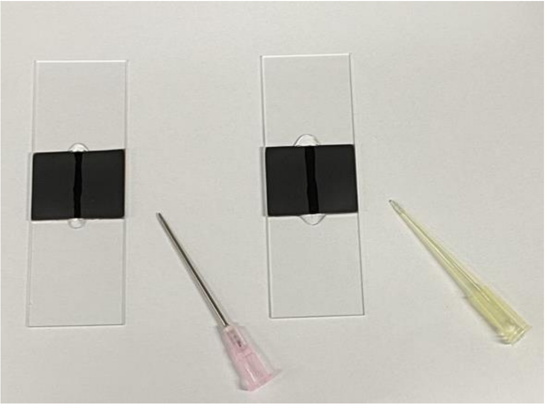

Supplementary Figure 5 | Photograph of the tips used to apply the epoxy.

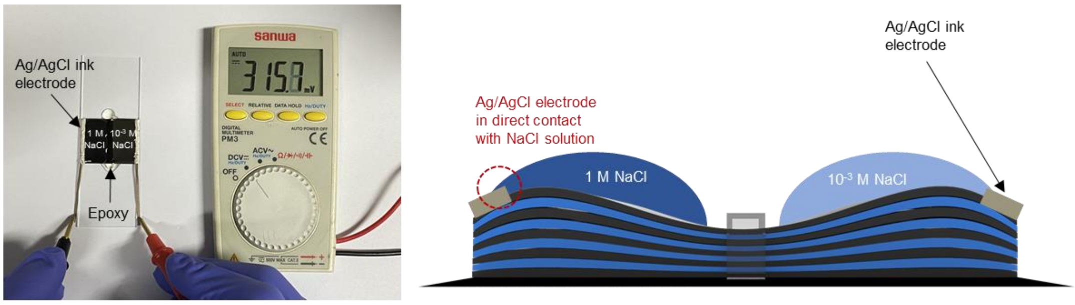

Supplementary Figure 6 | Photograph and schematic of unit cell when NaCl solution is dropped. The Ag/AgCl ink electrodes were pasted on both sides of the GO membrane. The structure allows direct contact between the electrode and the solution when the NaCl solution is dropped on the GO membrane.

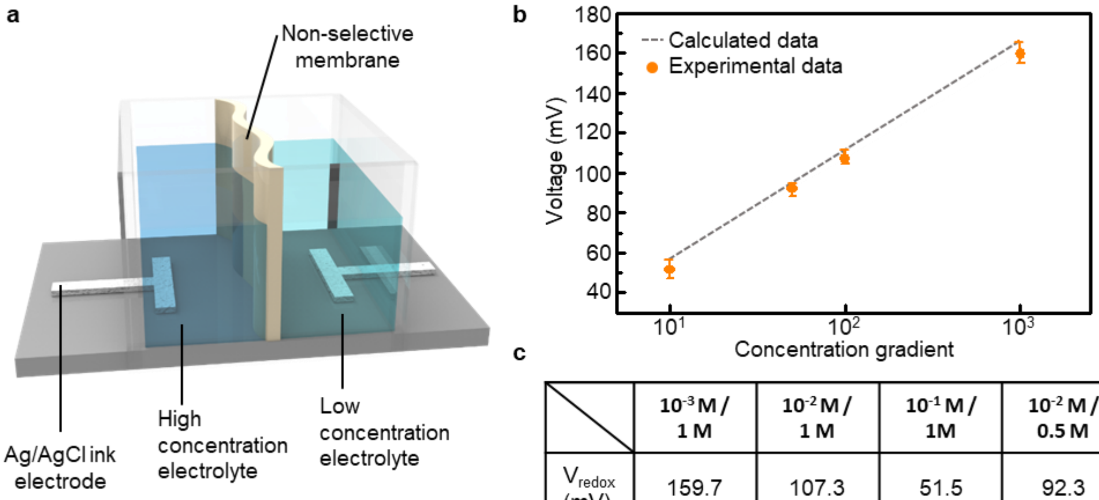

	10^{-3} M / 1 M	10^{-2} M / 1 M	10^{-1} M / 1M	10^{-2} M / 0.5 M
V_{redox} (mV)	159.7	107.3	51.5	92.3

Supplementary Figure 7 | Experimental setup for measuring *V*redox, and the corresponding measured data. a. Schematic of experimental setup for measuring redox potential of electrodes. Ag/AgCl ink electrodes were pasted and dried on the substrate. The solution was connected by a non-selective membrane (cellulose filter membrane of pore size 5 μm, Hyundai Micro Co., Ltd.). **b.** Comparison of calculated data and measured data of redox potential under various concentration gradients**.** Error bars denote standard deviation. **c.** Measured data of redox potential under various concentration gradients.

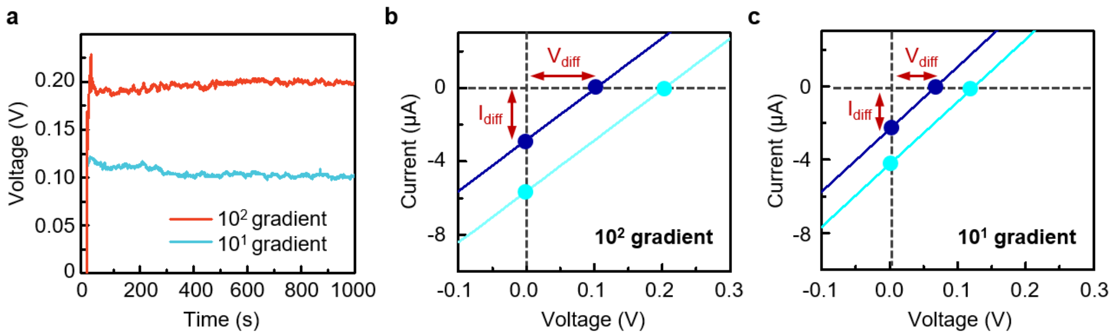

Supplementary Figure 8 | Voltage output data with time and *I*–*V* curve when concentration gradient is 10^1 and 10^2. a. Open circuit voltage over time under concentration gradients of 10^1 and 10^2. It is apparent that the ion selectivity was maintained under both the concentration gradients. **b.** *I*–*V* curve data under a concentration gradient of 10^2 across the channel. The value by ion selective diffusion (blue line) is obtained by subtracting the redox reaction of the electrode by the unequal chloride concentration at the measured value (sky blue line). V_{diff} and I_{diff} are the diffusion voltage and diffusion current, respectively. **c.** *I*–*V* curve data under a concentration gradient of 10^1 across the channel. Data indicates that ion selectivity is maintained above 90% under both the concentration gradients.

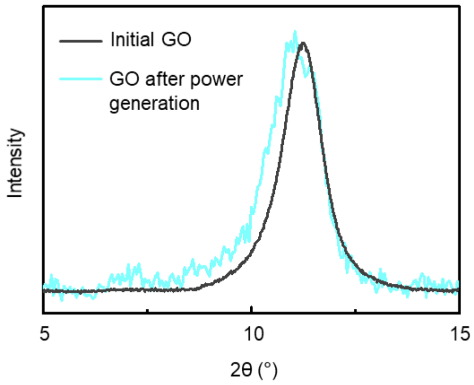

Supplementary Figure 9 | XRD pattern of GO channel part before and after the power generation.

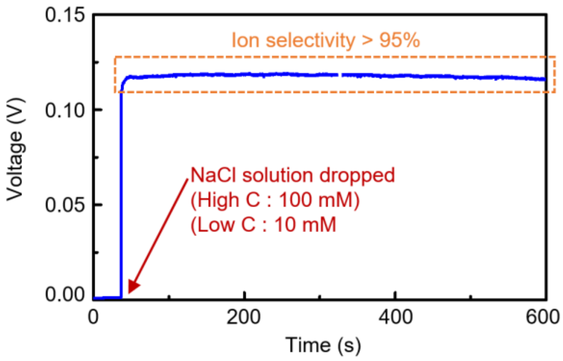

Supplementary Figure 10 | Open circuit voltage over time under the concentration gradient of 10^1 (100 mM/10 mM).

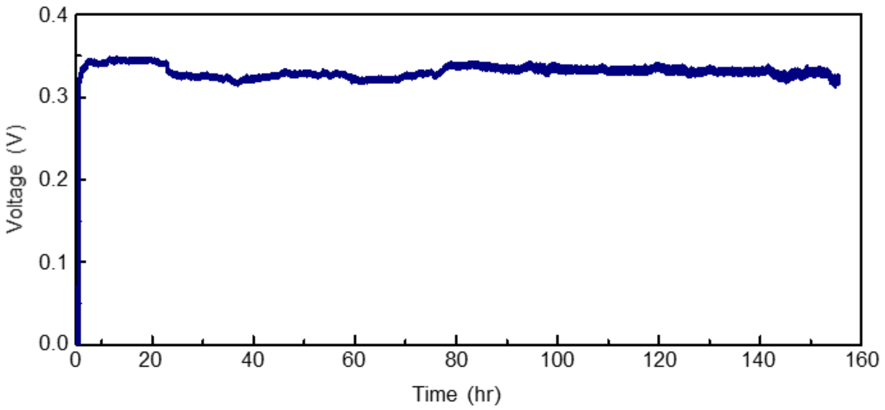

Supplementary Figure 11 | Open circuit voltage with respect to time under a concentration gradient of 10^3. The voltage output was measured in a sealed state so that the relative humidity was maintained over 70%.

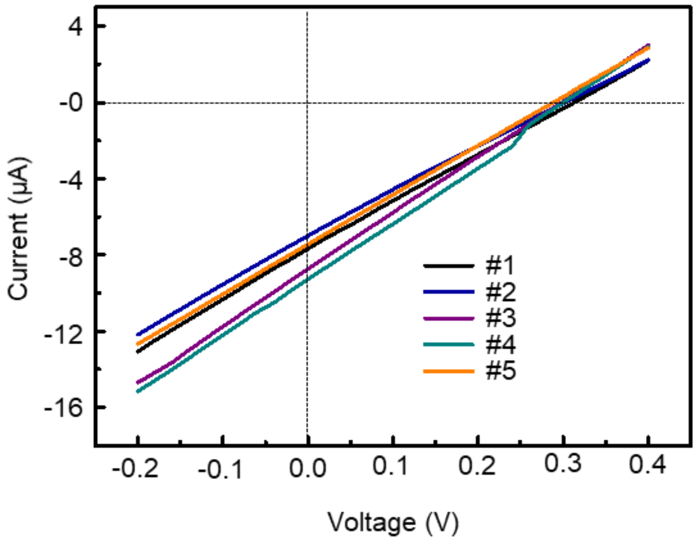

Supplementary Figure 12 | *I-V* curve data of 5 different cells (#1 ~ #5) under a concentration gradient of 10^3 at pH 7.

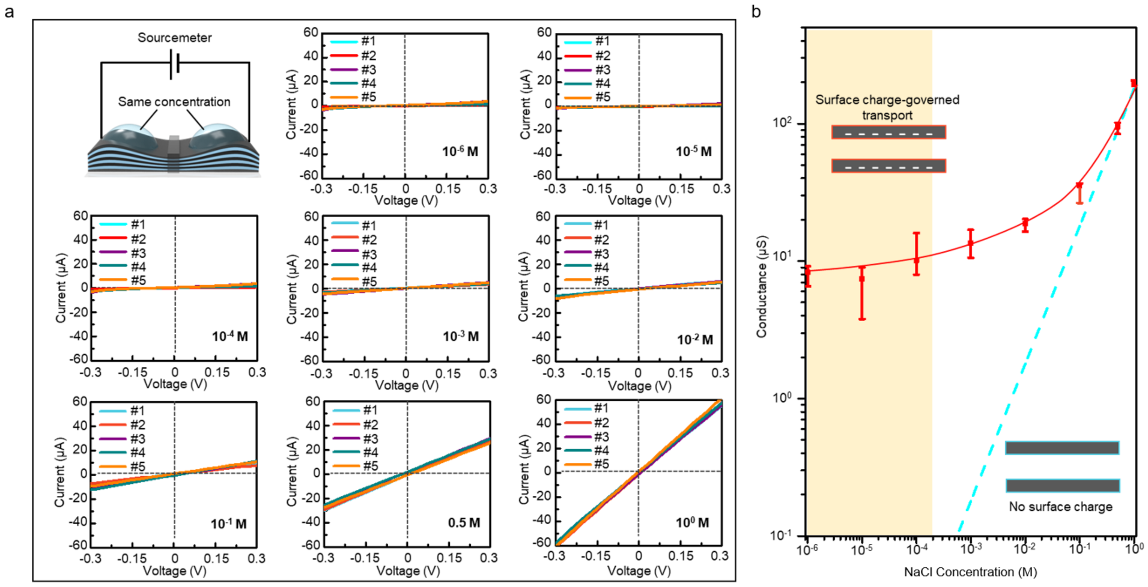

Supplementary Figure 13 | *I*–*V* curve of GO membrane according to the NaCl concentration of the solution, and conductance plot. a. *I*–*V* response at various concentrations. **b.** Ionic conductance as a function of NaCl concentration. The blue dashed line represents the ionic conductance of the ion transport without a surface charge (calculated value), and the orange full line represents the ionic conductance value (measured value) when a charge is present on the surface. Error bars denote standard deviation.

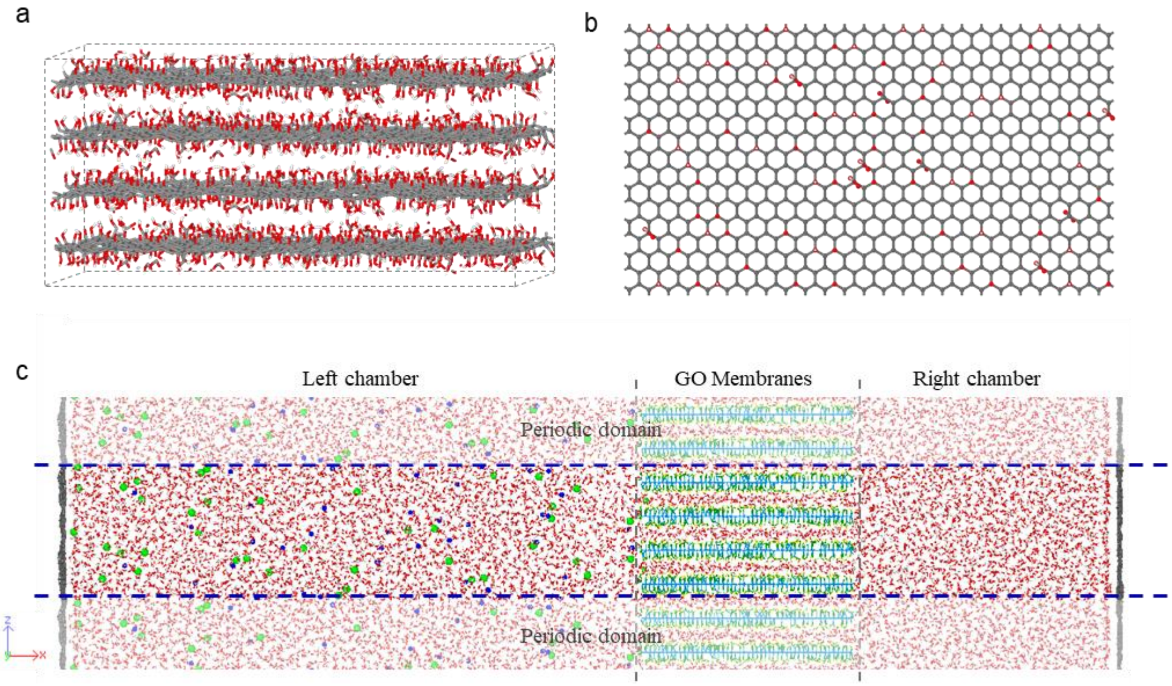

Supplementary Figure 14 | MD simulation setup for confined GO membrane. a. Initial atomic configuration for GO used in the MD simulations. **b.** The top view of an individual GO layer that is functionalized on both sides with the functional groups randomly distributed. **c.** Lateral view of the simulation system of the GO membrane, showing that the GO membrane was placed in the center of the box with two reservoirs on both sides of the membrane. The simulation box is marked between the two blue dashed lines. Carbon atoms in the GO sheets are shown as blue plates, oxygen as green spheres, hydrogen in yellow, Na^{+} in blue, Cl^{-} in green, water oxygen in red, and water hydrogen in white.

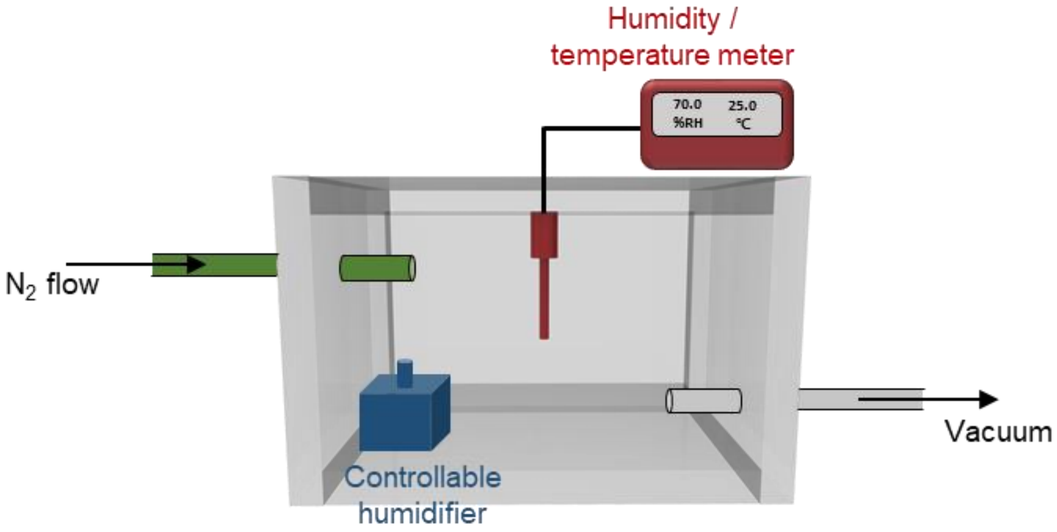

Supplementary Figure 15 | Schematic of humidity control setup.

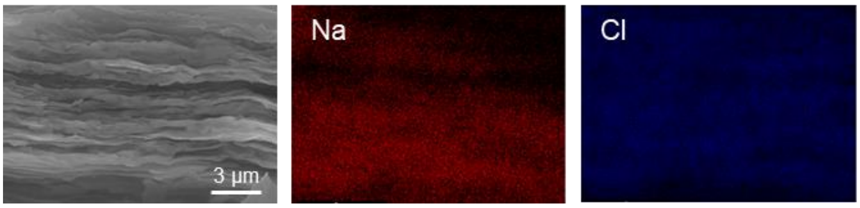

Element	Atomic %
Na	8.26
Cl	9.45

Supplementary Figure 16 | SEM image and EDS data of non-epoxy portion of GO membrane (reservoirs). It is apparent that the non-epoxy pasted part (reservoir) absorbed the NaCl solution, indicating that it exhibits low ion selectivity.

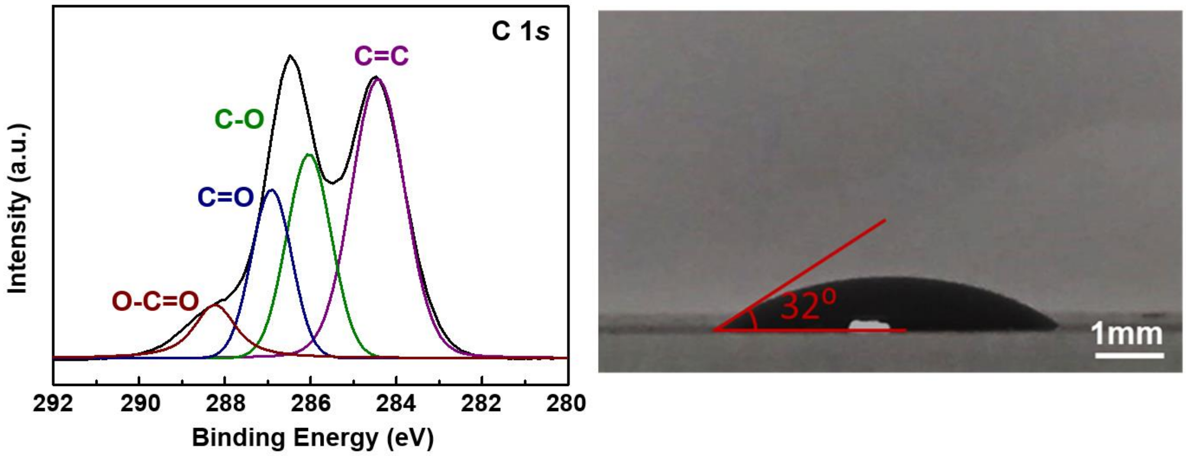

Supplementary Figure 17 | X-ray photoelectron spectroscopy (XPS) spectra and surface contact angle of GO. C 1*s* spectra were decomposed into four single peaks corresponding to four types of carbon bonds: C=C (284.4 eV), C–O (286.1 eV), C=O (286.9 eV), and O–C=O (288.2 eV).

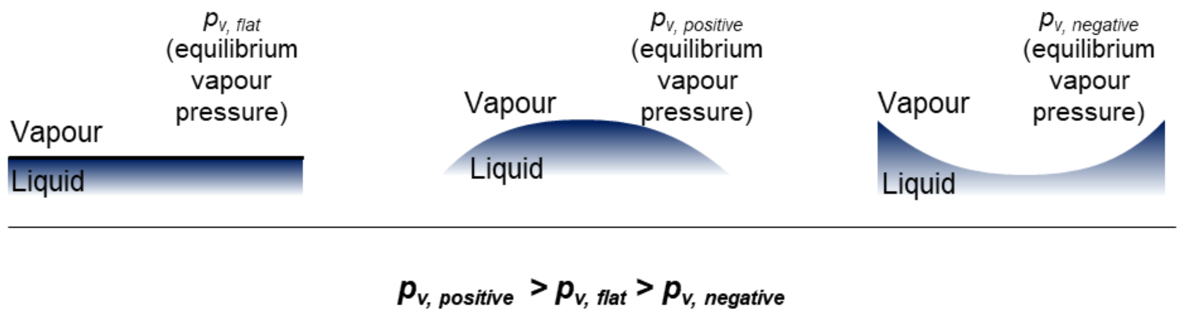

Supplementary Figure 18 | Vapour–liquid interface curvature dependence of equilibrium vapour pressure.

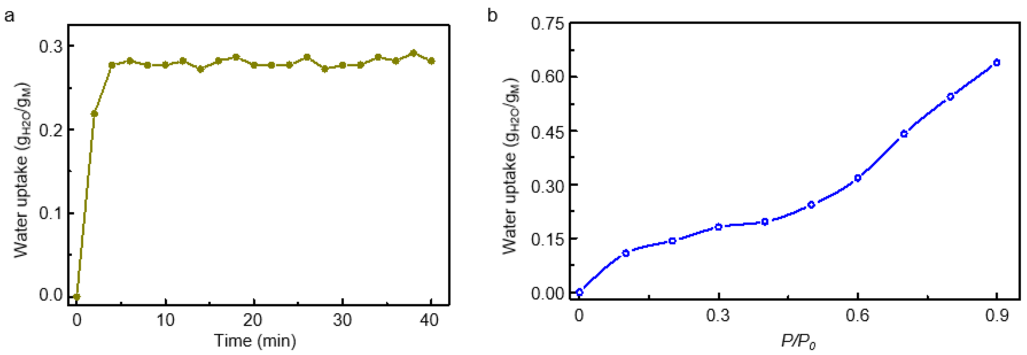

Supplementary Figure 19 | Water adsorption of GO. a. Water adsorption rate measured at 0.6 PP_0^{-1} and 298 K. **b.** Adsorption isotherms of GO membrane at 298 K.

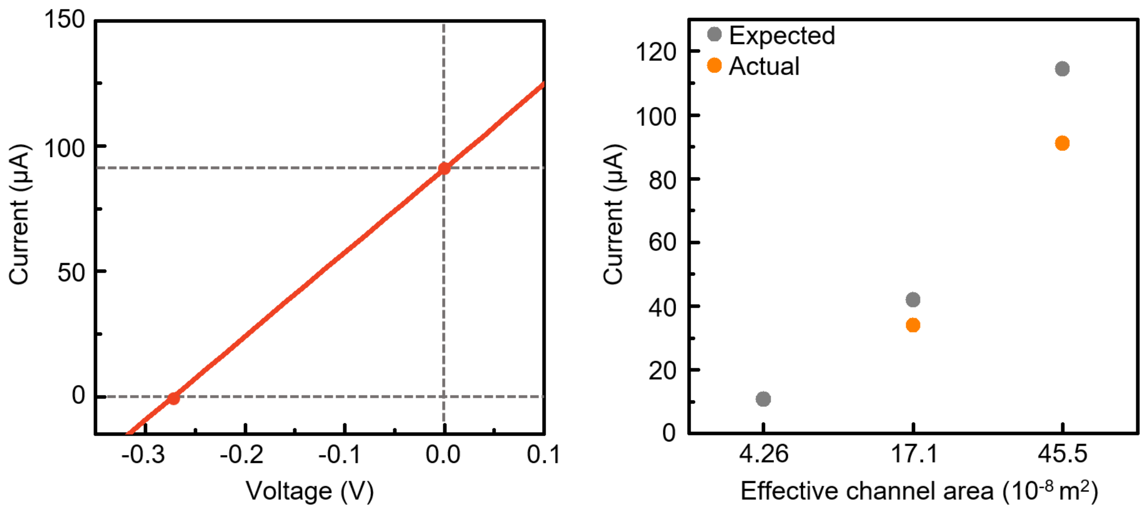

Supplementary Figure 20 | *I–V* curve when the effective surface area of the channel is 45.5 × 10^{-8} m^2 (10^3 concentration gradient), and short-circuit current with respect to the total cross-sectional area of the channel.

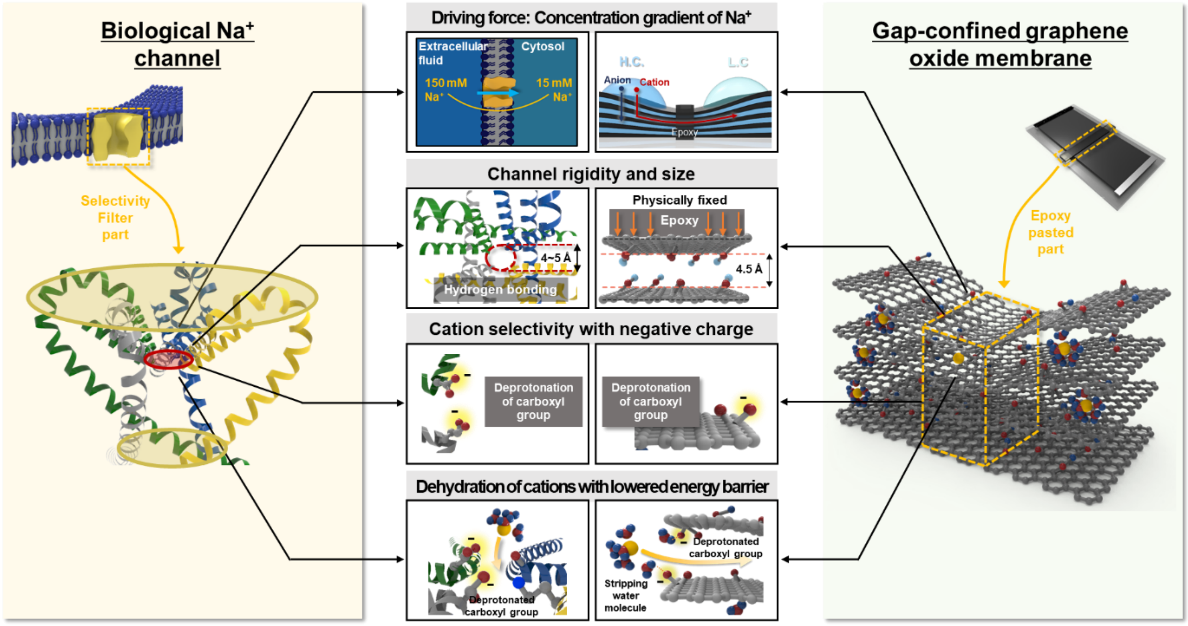

Supplementary Figure 21 | Schematic comparison of the neuron and confined GO membrane in the context of operation mechanism, structure, and charge effect for energy generation.

Supplementary Table 1 | Hydration enthalpy value according to ion species

Cation	Enthalpy of hydration (kJ)
Na^{+}	−405
K^{+}	−312
Ca^{2+}	−1565

Supplementary Table 2 | Comparison of performances with various materials

Type	Materials	Ion selectivity (%)	Energy conversion efficiency (%)	Power density (W/m^2)	Duration of the constant power generation (h)	Reference
**This work**	Graphene oxide	**95.8**	**41.4**	5.26	**160**	This work
**Two-dimensional materials**	Graphene oxide	73	26.6	0.77	16	Ref. 1
	Graphene oxide	-	-	0.35	-	Ref. 13
	Mxene	90	40.6	21	-	Ref. 2
	rGO	66	21	1.15	-	Ref. 14
	Mxene/Kevlar	80	35	4.1	-	Ref. 15
**Polymer**	PES-Py/PAEK-HS	74.6	35.7	2.66	120	Ref. 16
	PMA/PS-*b*-P4VP	-	13.2	3.8	-	Ref. 17
	Polyimide	60	4	0.26		Ref. 18
**Other materials**	Silicon	78.7	31	7.7	-	Ref. 19
	SNF/AAO	-	27.3	2.86	-	Ref. 20
	Hydrogel/ ANF	-	19.2	5.06	-	Ref. 21
	Mesoporous Carbon/AAO	80	37.3	3.46		Ref. 22

Supplementary Table 3 | Similarity and dissimilarity between biological Na^+ channel and confined GO membrane

		**Biological Na^+ channel**	**Gap confined GO membrane**
**Similarity**	**Driving force**	Concentration gradient	Concentration gradient
	**Reason of cation selectivity**	Deprotonation of carboxyl group	Deprotonation of carboxyl group
	**Channel rigidity**	○	○
	**Channel size**	4~5 Å	4~5 Å
	**Dehydration of ions**	○	○
	**Compensation of dehydration energy barrier**	Negative charge of carboxyl group	Negative charge of carboxyl group
**Dissimilarity**	**Channel length**	10~20 Å	3 mm
	**Channel shape**	Pore	Interlayer of two-dimensional materials

Reference

1. Ji, J. *et al.* Osmotic Power Generation with Positively and Negatively Charged 2D Nanofluidic Membrane Pairs. *Adv. Funct. Mater.* **27**, 1603623 (2017).

2. Hong, S. *et al.* Two-Dimensional Ti $_3C_2T_x$ MXene Membranes as Nanofluidic Osmotic Power Generators. *ACS Nano* **13**, 8917–8925 (2019).

3. Atkins, P. & Paula, J. de. *Physical Chemistry, 9th Edition*. (W. H. Freeman, 2009).

4. Esfandiar, A. *et al.* Size effect in ion transport through angstrom-scale slits. *Science* **358**, 511–513 (2017).

5. Plimpton, S. Fast Parallel Algorithms for Short-Range Molecular Dynamics. *J. Comput. Phys.* **117**, 1–19 (1995).

6. Chenoweth, K., van Duin, A. C. T. & Goddard, W. A. ReaxFF Reactive Force Field for Molecular Dynamics Simulations of Hydrocarbon Oxidation. *J. Phys. Chem. A* **112**, 1040–1053 (2008).

7. Chen, B., Jiang, H., Liu, X. & Hu, X. Molecular Insight into Water Desalination across Multilayer Graphene Oxide Membranes. *ACS Appl. Mater. Interfaces* **9**, 22826–22836 (2017).

8. Lorentz, H. A. Ueber die Anwendung des Satzes vom Virial in der kinetischen Theorie der Gase. *Ann. Phys.* **248**, 127–136 (1881).

9. Hockney, R. W. *Computer Simulation Using Particles*. (CRC Press, 1988).

10. Stukowski, A. Visualization and analysis of atomistic simulation data with OVITO–the Open Visualization Tool. *Model. Simul. Mater. Sci. Eng.* **18**, 015012 (2009).

11. Butt, H. *et al. Physics and Chemistry of Interfaces, 3rd Edition*. (Wiley, 2003).

12. Wei, N., Lv, C. & Xu, Z. Wetting of Graphene Oxide: A Molecular Dynamics Study. *Langmuir* **30**, 3572–3578 (2014).

13. Sun, P. *et al.* Realizing Synchronous Energy Harvesting and Ion Separation with Graphene

Oxide Membranes. *Sci. Rep.* **4**, 5528 (2015).

14. Wan, J. *et al.* Microwave Combustion for Rapidly Synthesizing Pore-Size-Controllable Porous Graphene. *Adv. Funct. Mater.* **28**, 1800382 (2018).

15. Zhang, Z. *et al.* Mechanically strong MXene/Kevlar nanofiber composite membranes as high-performance nanofluidic osmotic power generators. *Nat. Commun.* **10**, 2920 (2019).

16. Zhu, X. *et al.* Unique ion rectification in hypersaline environment: A high-performance and sustainable power generator system. *Sci. Adv.* **4**, eaau1665 (2018).

17. Zhang, Z. *et al.* Ultrathin and Ion-Selective Janus Membranes for High-Performance Osmotic Energy Conversion. *J. Am. Chem. Soc.* **139**, 8905–8914 (2017).

18. Guo, W. *et al.* Energy Harvesting with Single-Ion-Selective Nanopores: A Concentration-Gradient-Driven Nanofluidic Power Source. *Adv. Funct. Mater.* **20**, 1339–1344 (2010).

19. Kim, D.-K., Duan, C., Chen, Y.-F. & Majumdar, A. Power generation from concentration gradient by reverse electrodialysis in ion-selective nanochannels. *Microfluid. Nanofluidics* **9**, 1215–1224 (2010).

20. Xin, W. *et al.* High-performance silk-based hybrid membranes employed for osmotic energy conversion. *Nat. Commun.* **10**, 3876 (2019).

21. Zhang, Z. *et al.* Improved osmotic energy conversion in heterogeneous membrane boosted by three-dimensional hydrogel interface. *Nat. Commun.* **11**, 1–8 (2020).

22. Gao, J. *et al.* High-Performance Ionic Diode Membrane for Salinity Gradient Power Generation. *J. Am. Chem. Soc.* **136**, 12265–12272 (2014).
