# Supplementary material for: Neuromorphic van der Waals crystals for substantial energy generation
Source: Nat Commun. 2021 Jan 4;12:47. doi: 10.1038/s41467-020-20296-9 (PMC7782783; doi:10.1038/s41467-020-20296-9)
Supplement: Supplementary file 2 — Description of Additional Supplementary Files [file 41467_2020_20296_MOESM2_ESM.pdf]

Description of Additional Supplementary Files

Supplementary Movie 1
Switching on a single LED using a 6 series-connected cells.

Supplementary Movie 2
Switching on 19 LEDs using 16 series-connected cells.

Supplementary Movie 3
Switching on 19 LEDs using a commercial AA battery.
